# Impact of COVID-19 on the Changing Patterns of Respiratory Syncytial Virus Infections

**DOI:** 10.3390/idr14040059

**Published:** 2022-07-24

**Authors:** Ishan Garg, Rahul Shekhar, Abu Baker Sheikh, Suman Pal

**Affiliations:** Department of Internal Medicine, University of New Mexico, Albuquerque, NM 87106, USA; ishangargmd@gmail.com (I.G.); rshekhar@salud.unm.edu (R.S.); absheikh@salud.unm.edu (A.B.S.)

**Keywords:** respiratory syncytial virus, RSV, SARS-CoV-2, coronavirus 2019, COVID-19, RSV outbreak

## Abstract

Seasonal epidemics of respiratory syncytial virus (RSV) is one of the leading causes of hospitalization and mortality among children. Preventive measures implemented to reduce the spread of SARS-CoV-2, including facemasks, stay-at-home orders, closure of schools and local-national borders, and hand hygiene, may have also prevented the transmission of RSV and influenza. However, with the easing of COVID-19 imposed restrictions, many regions are noticing a delayed RSV outbreak. Some of these regions have also noted an increase in severity of these delayed RSV outbreaks partly due to a lack of protective immunity in the community following a lack of exposure from the previous season. Lessons learned from the COVID-19 pandemic can be implemented for controlling RSV outbreaks, including: (1) measures to reduce the spread, (2) effective vaccine development, and (3) genomic surveillance tools and computational modeling to predict the timing and severity of RSV outbreaks. These measures can help reduce the severity and prepare the health care system to deal with future RSV outbreaks by appropriate and timely allocation of health care resources.

## 1. Introduction

Seasonal epidemics of respiratory syncytial virus (RSV) is one of the leading causes of hospitalization and mortality among children due to bronchiolitis and pneumonia globally [1]. RSV is estimated to cause more deaths than any other infectious agent, except malaria [2]. According to a study by the Pneumonia Etiology Research for Child Health (PERCH) study group, RSV was the most common pathogen isolated from children (<5 years of age) hospitalized for severe pneumonia in Africa and Asia, accounting for 31% of total cases [1]. In a prospective population-based study in the United States (2015–2016 season), the RSV hospitalization rate was 2.9 per 1000 children <5 years of age and 14.7 per 1000 children <6 months of age [3]. RSV infection was associated with one-third of acute respiratory illness (ARI) hospitalizations in young children [3]. Globally, the estimated annual RSV-associated pneumonia mortality rate is 2.3% among neonates, rising to 6.7% of deaths among infants [2]. 

At present, there is no approved vaccine for use against RSV infection. However, various vaccine candidates are currently under investigation [4]. Therefore, measures preventing the spread of RSV remain the most promising means of controlling these seasonal epidemics. RSV is transmitted through inoculation of nasopharyngeal or ocular mucous membranes after direct contact with virus-containing secretions or fomites [5,6,7,8]. Interestingly, the coronavirus disease 2019 (COVID-19) pandemic, which is caused by severe acute respiratory syndrome-coronavirus-2 (SARS-CoV-2), shares a similar mechanism of transmission (exposure to infectious respiratory fluids). As a result, a broad range of public health measures implemented to control the spread of the COVID-19 pandemic, such as stay-at-home orders, school closures, travel restrictions and border closures, physical distancing, face masks, and other non-pharmaceutical interventions (NPIs), not only impacted COVID-19 but may have also changed the epidemiology of various other respiratory viruses, including RSV and influenza virus.

The purpose of this article is to review the current literature on the impact of the COVID-19 pandemic on RSV outbreaks and review limited data and theoretical considerations to predict and control future RSV outbreaks.

## 2. Impact of COVID-19 Pandemic on the Circulation of RSV

SARS-CoV-2 is transmitted through exposure to respiratory fluids carrying the infectious virus. Exposure occurs in three principal ways: (1) inhalation of very fine respiratory droplets and aerosol particles; (2) deposition of respiratory droplets and particles on exposed mucous membranes in the mouth, nose, or eye by direct splashes and sprays; and (3) touching mucous membranes with hands that have been soiled either directly by virus-containing respiratory fluids or indirectly by touching surfaces with the virus on them (fomites) [9,10,11,12,13,14]. Viruses affecting the respiratory tract including RSV and influenza are also transmitted through similar mechanisms [5,6,7,8]. Therefore, existing recommendations to prevent SARS-CoV-2 transmission include physical distancing, facemasks, stay-at-home orders, closure of schools and local-national borders, hand hygiene, and environmental cleaning may help prevent transmission of RSV and influenza virus as well [15]. 

Prior to the COVID-19 pandemic, RSV was one of the most common causes of lower respiratory tract infection in the pediatric population. The World Health Organization (WHO) piloted an initiative to establish RSV surveillance through the Global Influenza Surveillance and Response system [16]. The findings noted seasonal peaks in RSV activity, chiefly centered around the cooler months. Analysis of subtypes showed that different subtypes dominated in different seasons in each country. For example, RSV-A was the dominant strain in Argentina in 2017 and RSV-B was the dominant strain in Argentina in 2018. In the same period, RSV-B was the predominant strain in India in both 2017 and 2018. Among the countries reported in this study, Canada was the only nation to show co-circulation of both subtypes. The study also noted that the subtype of virus did not affect the timing of the epidemic. In the United States of America, RSV monitoring is performed through the National Respiratory and Enteric Virus Surveillance System (NREVSS). In the 2013–2014 season, 12% positive results were reported by eligible laboratories in this system. The national epidemic (excluding Florida) onset occurred in November, peaked in December–February and offset by April in most regions (Florida annually has an earlier onset in July and, therefore, was reported separately) [17]. In subsequent years, 2014–2017, onset was noted to occur in mid-October, with the peak in early February, and the offset by early May. It is important to note that these years note a change in the statistical method used to analyze data in NREVSS which may have affected the timing of the epidemic detection [18]. These findings of RSV circulation in pre-COVID-19 pandemic times provide a useful context to observe the changes in trends during the pandemic which are discussed subsequently.

In analysis of respiratory viral activity in United States during 2020–2021, the Centers for Disease Control and Prevention (CDC) reported historically low weekly percentage positive rate of RSV early in the pandemic (<1.0% per week compared to ~12–16% during peaks in previous years) [19]. Similarly, in Western Australia, RSV activity was noted to decline after COVID-19 restrictions were implemented (percentage positivity of test 0.28% vs. 23.3–30.4% in preceding years) [20]. In France, during the 2020–2021 season, a delayed peak of RSV epidemic was noted but the peak positivity during this season (11%) remained lower than the positivity during peaks in previous years (21–27%) [21]. Thus, it would be reasonable to conclude that circulation of RSV was decreased during the initial period of COVID-19 pandemic.

Although the restrictions imposed during the pandemic may be responsible for this decrease in circulation, what contribution each individual restriction, such as masking, social distancing, travel restrictions, school closures, etc., made remains to be seen. Some contexts may be provided by examining the temporal trends in relation to specific restrictions. In France, for example, the first lockdown included closure of schools and daycare whereas the second lockdown did not, and this was followed by a rise in RSV cases [21]. In Australia, a peak of RSV cases similarly followed easing of gathering restrictions and school re-openings but preceding relaxation of border restrictions, indicating perhaps the limited role of travel restrictions in the low circulation [20]. 

It is interesting to note that, in contrast to RSV, COVID-19 related restrictions had only a limited impact on rhinovirus circulation [22,23,24]. It is a non-enveloped virus and can survive on external surfaces for a longer duration than RS and SARS-CoV-2 viruses. This may partly explain the continued spread of rhinovirus during COVID-19-related restrictions. However, it is important to note that the exact underlying mechanisms remains to be explored.

## 3. Impact of the COVID-19 Pandemic on RSV Seasonality

RSV typically causes seasonal outbreaks between late fall and early spring. The timing of outbreaks may vary between different geographic regions and year to year. Examining surveillance data from individual countries shows certain patterns, The outbreaks usually occur from October–November to April–May in the northern hemisphere, peaking in January–February [19]. In the Southern hemisphere, these outbreaks typically occur from April–May to September–October, peaking in June–July [25]. In tropical and subtropical climates, the outbreaks are associated with rainy seasons, sometimes with persistent year-round incidence, leading to annual epidemics with shallow troughs [26,27]. This pattern of RSV seasonality by geography was also borne out in a systematic review by Stensballe et al. examining global trends [28]. This review examined 159 studies and found that in equatorial regions, RSV can be detected throughout the year, though with seasonal peaks in incidence with a more temporal trend as one moves further toward the temperate regions. A trend was also noted in RSV seasonality in relation to distance from the coast, with RSV starting in coastal areas and appearing to move inward in subsequent months. This was theorized to be not due to contiguous spread but rather, in part, due to weather dependent behaviors, such as indoor crowding. Similarly, different parts of a country may experience differences in seasonality which may be based on local climate. For example, the south of the United States of America experiences an earlier and longer epidemic than the mid-west region of the United States of America [29].

The initiation of COVID-19 preventive measures and travel restrictions in 2020 coincided with the end of winter in the Northern hemisphere and the start of winter in the Southern hemisphere (start of RSV season) [30]. Studies from the Southern hemisphere noted a drastic reduction in new RSV cases [20] compared to previous seasons. For instance, an Australian (western-Australia) study found 98.0% and 99.4% reductions in RSV and influenza detections, respectively, in children during the winter of 2020 [30]. Similar findings are reported from the northern hemisphere, suggesting interruption of RSV transmission during the winter of 2020/2021. For instance, a Japanese study noted a reduction of approximately 85% in the average number of monthly RSV cases in 2020 when compared to those in the preceding six years (2014*–*2019) [31]. In a Korean study, based on data from Respiratory Virus Monitoring System, they reported a significant reduction in positive rates of RSV to 19% of predicted value compared to the pre-non-pharmaceutical interventions (NPIs) period [32]. They also noted a significant reduction in hospitalization to 9% of the predicted value. Similar results were observed in a Chinese study, where RSV infections decreased dramatically from 4.64% (95% CI, 2.88–7.64) in previous years to 0.0% in 2020 [33]. Studies from European countries also showed a drastic decrease in RSV cases during the usual RSV epidemic season starting from March 2020 (coinciding with COVID-19 related measures), compared to the preceding years. In Italy, RSV was noted to be a common respiratory isolate in December 2019 to January 2020, but virtually disappeared following the appearance of SARS-CoV-2 in this country [34]. Another Italian study noted an average 84% reduction in the rate of acute bronchiolitis (of which RSV is a common cause) in pediatric population in 2020–2021 compared with the previous five years (2015*–*2019) [35]. A study of respiratory tract infections in children in Finland showed that RSV infection rates tracked the pattern of previous years in the first 12 weeks of 2020 but then abruptly declined, coinciding with timing of nationwide lockdown [36].

However, several southern and northern hemisphere countries are now reporting delayed RSV peaks in the spring and summer following the lifting of COVID-19 pandemic-imposed restrictions [29]. It is also important to note that various additional factors, including climate, population behavior are also important drivers of RSV seasonality. For instance, meteorological factors, diurnal temperature range, precipitation and relative humidity were noted to be associated with increased RSV transmissibility, and higher maximum wind speed was associated with decreased RSV transmissibility [37]. It may be theorized that meteorological factors may influence RSV seasonality through either direct effect on circulation or through alterations in human behavior (such as increased congregation indoors in cold or wet months) but exact mechanisms remain unknown and a detailed discussion about these are beyond the scope of this article.

A similar pattern of RSV seasonality was noted following the 2009 influenza pandemic [38,39]. In a global-level systematic analysis, Li et al. pointed out that the influenza pandemic delayed the onset of the first RSV season by 0.58 months (95% CI: 0.42, 0.73) and the start of the second RSV season by only 0.25 months (95% CI: 0.12, 0.39); with no delayed onset observed for the third RSV season. The delayed onset was most pronounced in the northern temperate region, followed by the southern temperate region, and was least pronounced in the tropics [40].

These regional variations in the impact of the influenza pandemic on RSV seasonality could be due to relative differences in the timing of the influenza pandemic and timing of the regional RSV season. For example, in the southern temperate region, the onset of RSV season (April–May) coincided with the start of the influenza pandemic. Although in the northern temperate region, the onset of RSV season (October–November) occurred when the influenza pandemic was already well underway. By comparison, the timing of RSV season is more varied in the tropics and, therefore, less impacted by the timing of onset of the influenza pandemic.

### A Delayed Outbreak of RSV

Surveillance data from various countries have shown delayed RSV outbreaks during the 2020*–*2021 season [41]. In a surveillance study from Europe, including 17 European countries, using the European Centre for Disease Prevention and Control (ECDC) surveillance atlas data, Summeren et al. noted that circulation of RSV stopped after NPIs were introduced to control SARS-CoV-2 circulation in February–March 2020 [40]. RSV epidemics were only observed in France and Iceland for the 2020/2021 winter, with epidemics starting several weeks later than usual.

They also noted that since mid-May 2021, RSV outbreaks had been noted in several European countries, including Sweden, Belgium, Netherlands, Italy, Spain, Portugal, and Denmark, as well as Japan, Israel, and India, with a significant delay in onset compared with previous seasons 2016/17 to 2019/2020 [41,42,43,44,45,46,47]. For instance, in the Netherlands, a very late out-of-season RSV epidemic started in week 24, 2021, compared with weeks 46 and 48 in previous seasons (delay of at least 28 weeks). In France and Iceland, delays in the start of the RSV epidemic by at least 12 and 4 weeks, respectively, compared to previous seasons were noted [40].

In the southern hemisphere, surveillance data from Australia, Argentina, Brazil, Chile, New Zealand, and South Africa showed no RSV outbreak in the winter (June to September) of 2020/2021 [48,49,50,51,52,53,54,55]. The delayed onset of the RSV epidemic was noted at the beginning of the spring season in Australia, South Africa, Chile, and Argentina [54]. In addition, it has been noted that the RSV season in Australia (4 months in 2020 compared to 6 months in 2016*–*2019) and South Africa (5 weeks compared to 19*–*33 weeks in 2009 to 2016) was much shorter compared to previous seasons [53,55].

## 4. Impact of COVID-19 on RSV Epidemic Size

### Immunity Debt

As regular life resumes following the easing of COVID-19 pandemic-related restrictions, the exposure to various infectious viruses and bacteria—such as influenza, chickenpox, and RSV—will increase as well. However, the question remains on the severity of these new RSV epidemics occurring soon after a year of low activity. Immunity debt is defined as a lack of immune stimulation and protective immunity due to reduced exposure to a given pathogen, leaving a greater proportion of the population susceptible to the given pathogen in the future [56,57,58,59,60]. Immunity debt involves a complex mechanism involving interplay of innate and adaptive immunity (including training of innate immunity, development of immune memory), intestinal microbiota, exposure to pathogens, and immunization [61].

Some authors have suggested a combination of increased exposure following re-opening of schools, nurseries, and kindergartens, easing of travel restrictions, and cessation of mandatory facemask-wearing (non-pharmaceutical interventions) following COVID-19 vaccination, might mean that the future RSV outbreak might be more severe compared to previous seasons [21,62,63,64]. For instance, data from Japan showed that following no outbreak of RSV in 2020, the largest annual increase in RSV cases (since monitoring began in 2003) was reported for 2021 [63]. Similar findings were reported from Israel, where they found a higher peak (with higher weekly case numbers and incidence rates) in spring/summer of 2021 following a low incidence of RSV infections in 2020 [45]. In the southern hemisphere, supportive findings were observed in certain regions in Australia. An RSV-free winter was followed by a delayed RSV outbreak with a steeper peak affecting a population with older median age [51]. However, contradictory reports from other regions, including France, noted a shallower peak during the delayed RSV epidemic [54]. These findings highlight the complex phylogeographic dynamics, complex factors affecting RSV seasonality, viral genomic variability, viral genotype, and host phenotype interactions, as well as the challenges in predicting the behavior of future RSV outbreaks.

## 5. RSV and COVID-19 Co-Infection

COVID-19 and RSV can present with overlapping symptoms in children. However, the treatment of infections caused by these viruses is different. To make matters even more complicated, some patients can present with a co-infection with RSV and COVID-19 [65,66,67,68]. In a study on 713 pediatric patients hospitalized for COVID-19 infection, a viral coinfection rate of 15.8% (of which 66.4% had RSV infection) was noted [69]. However, the study did not include analysis on impact of co-infection with RSV and COVID-19 on clinical course and illness severity. By contrast, in a study by Lin et al. who, in a retrospective study including 81 COVID-19 positive children, noted RSV co-infection in only 1 patient (1/81) [70]. Similar findings were reported by Uhteg et al. on a cohort of 179 SARS-CoV-2 positive patients [71]. The authors found 0 patients with co-infection by other respiratory pathogens, including influenza and RSV. The author suggested this low rate of co-infection may, in part, be due to impact of COVID-19 pandemic on the circulation of certain respiratory viruses.

In a study by Choudhary et al. [72] on COVID-19-related pediatric hospitalizations, including 759 COVID-19 positive patients, an age-related trend was noted for RSV co-infection (42 (23.9%), 26 (21.3%), 4 (2.8%), and 3 (1.1%), in infants, children aged 1–4 years, 5–11 years, and 12–17 years, respectively). The authors also noted the co-infection with RSV to be one of the factors associated with severe illness (prevalence ratio of 3.64, 1.96, 2.2, and 2.48, in infants, children aged 1–4 years, 5–11 years, and 12–17 years, respectively). The severe illness was defined as patients receiving high-flow nasal cannula, positive airway pressure, or invasive mechanical ventilation. Similar findings were reported by Inger et al. in a prospective study involving 341 hospitalized children with common human coronavirus (cHCoV) [73]. The authors noted that patients with cHCoV-RSV co-infection had a severe disease (with adjusted odds ratio, 3.3; 95% confidence interval: 1.6–6.7) and higher risk of development of LRTI than in patients with single cHCoV infection.

With the ongoing pandemic of COVID-19 and the resurgence of RSV epidemics, it is possible that co-infections of these respiratory viruses may continue to occur. Findings from the literature noted above suggest a trend to more severe disease in co-infection, an effect that also showed an age-related trend, with younger COVID-19 positive at increased risk for RSV co-infection and more severe clinical course. However, little is known about several aspects of care for patients with co-infection. Robust data on the impact of co-infection on mortality or long term sequalae is lacking. Similarly, while effective post exposure prophylaxis is available for RSV and COVID-19, their effectiveness in cases with co-infection is unknown. Early detection, appropriate monitoring and reporting of data will be essential to answer these. Rapid diagnostic tools to detect and differentiate these viral agents in upper respiratory tract specimens may be a first step in pursuit of this. Some of these kits with the ability for qualitative detection and differentiation of SARS-CoV-2, Influenza A&B, RSV have shown promising results, such as Xpert Xpress SARS-CoV-2/Flu/RSV (Xpert 4-in1) assay; Xpert Xpress SAR S-CoV-2/Flu/RSV; PowerChek SARS-CoV-2, Influenza A&B, RSV Multiplex Real-time PCR Kit; and BioFire Respiratory Panel 2.1 [74,75,76,77].

## 6. Future Directions: How to Be Prepared for Potential Upcoming Resurgence

### 6.1. Public health interventions

Multiple public health measures were implemented during the COVID-19 pandemic to slow the spread of SARS-CoV-2, including physical distancing, facemasks, hand hygiene, and environmental cleaning. Similar measures can be used to control the spread of RSV pandemics. Examination of the effects of individual measures, when possible, can help determine efficacy in local and regional context. The development of large-scale surveillance data tools is needed to improve understanding of the disease and allow better modeling for resource allocation and healthcare policies, including timing and effectiveness of prophylactic and therapeutic interventions. The COVID-19 pandemic provided a rare moment in time when public health interventions were adopted on a massive scale for a limited time. A detailed regional analysis of temporal trends in RSV infections compared to previous years around the time of implementation and lifting of specific interventions, such as mask mandates, school closures, and travel restrictions, can provide valuable insight into the most effective strategies to prevent/mitigate future epidemics of RSV in each local context.

### 6.2. Research opportunities

RSV genomics—Lessons learned from the COVID-19 pandemic can be transmuted and implemented to control RSV outbreaks, including the large-scale surveillance and collection of RSV genetic data. Several ongoing studies have already examined RSV genomics [78,79,80,81,82,83,84,85]. However, a unified global effort is needed to establish a uniform approach to vital genotyping [83,84,85]. A better understanding of RSV genomics may help improve our understanding of disease epidemiology, track transmission patterns, identify reservoirs of infection, and identify targets for the development of effective vaccines [86].

Prophylactic Interventions — Multiple potential vaccines and therapeutic interventions are currently under development, including live-attenuated, chimeric, vector-based, and subunit-based vaccines [86]. However, as seen during COVID-19 pandemic, vaccine hesitancy can be a challenge that will need to be considered and mitigated for effective vaccination efforts.

### 6.3. Clinical Implications

COVID-19 and RSV co-infections—It is essential to be aware of co-infections in RSV-positive patients. COVID-19 and RSV co-infection in children may present with overlapping symptoms. Therefore, it is essential to develop rapid diagnostic tools to detect and differentiate these viral agents in upper respiratory tract specimens. Some studies have suggested that patients with COVID-19 and RSV co-infection may be at increased risk for severe clinical illness and may need respiratory support for hypoxemia, ranging from supplemental oxygen and non-invasive ventilation to intubation and mechanical ventilation. However, much remains to be known about the disease patterns in patients with co-infection, and reporting of such cases will be essential to fill the knowledge gap. Particular attention would also be needed on the impact of efficacy of post-exposure prophylaxis (for RSV and COVID-19) and vaccination (for COVID-19) on the disease course in patients with co-infection.

Immunity debt—The increase in the pool of people susceptible to respiratory pathogens, including RSV, due to measures implemented to restrict the spread of COVID-19, combined with a change in RSV seasonality, may make it difficult to predict the scale and time of onset of next RSV pandemic. Therefore, a higher clinical suspicion and close surveillance of trends may allow early diagnosis, treatment, and timely implementation of preventative measures, which may help prevent and contain the spread of a future RSV pandemic.

Therapeutic interventions—At present, only palivizumab (monoclonal antibody), a monoclonal antibody against the RSV fusion protein, is available for prophylaxis in high-risk patients, including infants with underlying comorbidities, such as bronchopulmonary dysplasia or congenital cyanotic heart disease [87,88,89]. Palivizumab is usually administered at monthly intervals during the 5–6 months of the peak RSV season. However, recently the American Academy of Pediatrics issued guidance supporting consideration for the use of palivizumab in eligible patients during the inter-seasonal spread of RSV (in regions experiencing high rates of RSV circulation in the spring and summer of 2021) [90]. For the current fall and winter season, the AAP recommends administering palivizumab prophylaxis in all country regions at the usual time, regardless of whether an area experienced unusual inter-seasonal RSV activity. Whether eligibility will need to be expanded to other cases, such as in co-infection with COVID-19, remains to be seen.

## 7. Limitations

This review has several limitations. Due to multiple measures implemented during the COVID-19 pandemic, it is not feasible to identify the impact of these individual measures on RSV epidemiology. Furthermore, due to the lack of consistent information on the heterogeneity of RSV genomic lineage in various geographical regions, it is impossible to predict the magnitude or length of the RSV outbreaks.

## 8. Conclusions

Public health interventions for countering the spread of the COVID-19 pandemic significantly impacted the transmission of other respiratory viruses, including RSV. This has led to minimal or no outbreak during the usual season of RSV (winter season in temperate climates). These findings also highlight the impact of effective Infection Prevention and Control (IPC) measures to reduce future RSV outbreaks. However, with easing of COVID-19 related restrictions, this lack of immunity due to the absence of RSV exposure may have increased susceptibility of the unexposed population to a more severe outbreak in future seasons. Information on change in RSV seasonality is vital for timely implementation of appropriate interventions, including administration of RSV prophylaxis (palivizumab) in high-risk populations, as well as the preparedness of health care systems to deal with any future outbreak.

## Data Availability

Not applicable.
